# The Effectiveness of Shared Care in Cancer Survivors—A Systematic Review

**DOI:** 10.5334/ijic.3954

**Published:** 2018-10-12

**Authors:** Yan Zhao, Alison Brettle, Ling Qiu

**Affiliations:** 1 Department of Urology, Minimally Invasive Surgery Center, The First Affiliated Hospital of Guangzhou Medical University, Guangzhou 510230, CN; 2 School of Nursing and Midwifery, University of Salford, Salford, Greater Manchester, M5 4WT, UK; 3 Minimally Invasive Surgery Center, The First Affiliated Hospital of Guangzhou Medical University, Guangzhou 510230, CN

**Keywords:** shared care, survivors, follow-up, cancer

## Abstract

**Objectives:**

To determine whether the shared care model during the follow-up of cancer survivors is effective in terms of patient-reported outcomes, clinical outcomes, and continuity of care.

**Methods:**

Using systematic review methods, studies were searched from six electronic databases—MEDLINE (n = 474), British Nursing Index (n = 320), CINAHL (n = 437), Cochrane Library (n = 370), HMIC (n = 77), and Social Care Online (n = 210). The review considered all health-related outcomes that evaluated the effectiveness of shared care for cancer survivors.

**Results:**

Eight randomised controlled trials and three descriptive papers were identified. The results showed the likelihood of similar effectiveness between shared care and usual care in terms of quality of life, mental health outcomes, unmet needs, and clinical outcomes in cancer survivorship. The reviewed studies indicated that shared care overall is highly acceptable to cancer survivors and primary care practitioners, and shared care might be cheaper than usual care.

**Conclusions:**

The results from this review suggest that the patient satisfaction of shared care is higher than usual care, and the effectiveness of shared care is similar to usual care in cancer survivorship. Interventions that formally involve primary care and improve the communication between primary care and hospital care could support the PCPs in the follow-up.

## Introduction

With the improvements in medical treatment and ageing populations, the number of cancer survivors is increasing worldwide [1,2]. Cancer survivors are vulnerable to suffering from second cancer and comorbid chronic conditions with advanced age [3,4]. Historically, most cancer patients were followed up by hospital specialists [5,6]. However, Nielsen et al. [7] believes that most cancer patient might feel left alone after they are discharged from the hospital. Besides, Yang et al. [8] argues that cancer specialists might not be able to provide necessary care unless there is enhanced oncological productivity. Due to the increased need, stabilised health care costs, and the sustainable burden of hospitals, the involvement of primary care has been increasingly recognized as a vital component in the management of cancer survivors [9,10].

To date, much of the studies mainly assessing the capability of primary care providers (PDPs) in survivorship care and indicate that the PDP’s skills and confidence may be lacking, but their skills could be enhanced by collaboration with hospital specialists [11,12]. In addition, a landmark report from the US Institute of Medicine lists coordinative care between primary care and secondary care as an essential component for cancer survivorship care [13]. Shared care that integrate primary care and hospital care was originally created for patients with chronic disease [14], and it has been endorsed as an important component of high-quality of survivorship care [15]. Johnson et al. [16, p. 350] defines shared care as:

“an organizational model involving both primary care physicians (PCPs) and specialists in a formal, explicit manner.”

Shared care does not only mean that both hospital and primary care join in the follow-up, but also means there is interaction between them. It is argues that the key points of this model are the communication between the care providers by exchange of information and arranging responsibility to improve the follow-up management [17].

The published reviews have focused on comparing the primary care provider and cancer specialist in the management of cancer follow-up. Lewis et al. [5] released a systematic review that aimed to evaluate the effectiveness and cost-effectiveness of cancer follow-up by primary care. The author could not make a conclusion since the quality of data was generally poor and no statistically significant difference was found in the effectiveness of primary care follow-up. A second review [18] argued that local health care practitioners could benefit patients with physical and psychosocial problem in survivorship care, but proactive initiatives should be conducted to involve PDPs in the follow-up. However, although integrating PCPs into the survivorship care is needed, recent reviews found little evidence regarding the effectiveness of shared care, and there is a lack of standard models of shared care [6,10,14,19].

## Review questions and objectives

In this study, we systematically review the literature that focuses on the effectiveness and feasibility of shared care in the management of follow-up for cancer patients in different settings, and critically appraise the quality of evidence. The key objectives are: 1) to evaluate whether shared care is feasible or effective in the management of physical or psychological problems in cancer survivors; 2) to provide a comprehensive review of the studies for achieving best practice in the management of follow-up for cancer survivors. The primary outcome will be whether shared care could solve the survivors’ physical or psychological problems, and patients’ attitudes toward shared care will be summarised. The physical problem could include the quality of life, the side effect, the recurrence rate, or any other symptoms. The psychological problems would include the anxiety, the distress level or other mental health disorders. The secondary outcomes include the patient reported and practitioner reported satisfaction towards shared care, and the cost of shared care. Studies that assessed shared care in short- and long-term cancer survivors were both included and reported, but those assessed patients at the end of life were not included because they usually need more complicated care and a lot of them might stay in the intensive care unit or hospice care unit [20].

## Methods

### Search strategy

The PRISMA systematic review and meta-analysis protocols (PRISMA-P) [21] was used as the guideline in this study, although minor changes were made to adapt to unanticipated circumstances. Six databases were searched based on the research question and objectives of this study—MEDLINE (Ovid), British Nursing Index, CINAHL (EBSCO), Cochrane Library, Health Management Information Consortium (HMIC), and Social Care Online. Besides, two journals—*Journal of Clinical Oncology* and *Journal of Adolescent and Young Adult Oncology* were identified for hand searching, and all reference lists of the selected papers and relevant reviews were looked through. The search terms were identified based on the planned population, intervention, comparison, and outcome (PICO) [22], and they were adjusted slightly according to the different databases (see Appendix 1). Table 1 shows the core components of the search strategy, and the last search was conducted on 17^th^ May 2017.

**Table 1:** Core components of the search strategy.

Population	Intervention	Context	Outcome

cancer (MeSH)	shared care (MeSH)	follow-up (MeSH)	All outcomes are included.
neoplasms (MeSH)	co-management	After care (MeSH)	
cancer*	“sharing of care”	aftercare	
neoplas*	“collaborative care”	follow up	
malignan*	“care coordination”	followup*	
carcinoma*	“coordinated care”	postsurgery	
sarcoma	“referral and consultation”	post-surgery	
oncolog*	“cooperative behavio?r”	postsurgical*	
tumo?r*	“shared service*”	post-surgical*	
adenocarcinoma*	“delivery of health care”	postoperat*	
infiltrat*	“integrated care”	post-operat*	
medullary	“shared model”	“continuity of patient care”	
intraductal	“inter-organizational coordination”	“disease management”	
		surveillance	
		“disease progression”	
		survivorship	
		rehabilitation	
		post treatment	
		post-treatment	
		posttreatment	

### Eligibility criteria

Inclusion criteria: 1) all types of primary research studies which assesses the shared care model in the management of follow-up for cancer patients, the formal interaction between primary care and secondary care; AND 2) include studies that examine any outcome in all types and any stage of cancer; AND 3) the population of interest included cancer survivors in any age; AND 4) published in English.

Exclusion criteria: 1) there was no formal interaction between primary care and secondary care as it is not shared care; OR 2) articles without outcomes such as commentary, protocol, or meeting abstract; OR 3) the research did not report any outcome about shared care; OR 4) the healthcare service were provided by other practitioners rather than hospital specialists and primary care team, or a multidisciplinary team include other practitioners; OR 5) the patients did not finish all the curative intent or adjuvant treatment; OR 6) the study only focus on the transition manner rather than the whole follow-up process.

### Selection process and method of appraisal

The author screened the title and abstract first, and any study that seemed to meet the selection criteria was full-text screened. Besides, two other reviewers randomly selected and reviewed 20% of the search records independently. Subsequently, studies were picked out according to the inclusion criteria. Any discrepancies were discussed and where there was any uncertainty, the professor with experience in systematic reviews was consulted. In addition, the study author was contacted by email when more information was needed. The reasons for excluding the studies were recorded to enable transparency of the selection process.

The Critical Appraisal Skills Programme (CASP) is a widely available appraisal tool developed by Oxford University, which includes eight checklists for the different types of studies [23]. The RCTs were appraised by the CASP Randomised Controlled Trial Checklist (see Appendix 2). The CASP comprises of 11 questions that assist a systematic thinking about the paper. The Health Care Practice R&D Unit (HCPRDU) has developed three checklists, with 6, 6, 7 sections respectively, to appraise quantitative, qualitative and mixed-method studies [24]. Thus, other studies were appraised with one of the HCPRDU checklists according to the research methodology (see Appendix 3, 4). Häggman-Laitila et al. [25] evaluated the studies by selecting a score from 0 to 2 points in a systematic review, and this method was utilised and adapted for this review. For all studies, each question on the appraisal tools was scored from “0” to “2” separately and then the total scores were calculated. Among them, “0” means many limitations, “1” means some limitation, and “2” means excellent.

### Data collection and synthesis of the findings

Gough et al. [26] argue that the content of both quantitative and qualitative studies should be described and coded first, then the data extracted from these descriptions can be synthesised into the findings. Therefore, the thematic analysis, which is a widespread analytical method to accommodate a diversity of studies including both experimental and observational studies [27], was used in this review. Besides, since the data in the selected studies are not sufficiently similar to allow for meta-analysis, the narrative approach was utilised in this review [28]. First, one author extracted the information and the other author verified all the information, the disagreements were solved by discussion; second, after a comprehensive understanding of all the results and a critical scrutiny of the themes was achieved, the final theme was summarised from all the papers in order to answer the research question; finally, the sample size, the study design, the limitation, and the quality of evidence were considered when there were conflicting themes, and the study with larger sample size or better quality might contribute more to the conclusion. The main review focus on the objectives of the systematic review, and the subgroup analysis were also included when they important to answer the questions of the review questions.

## Results

**Figure 1:**
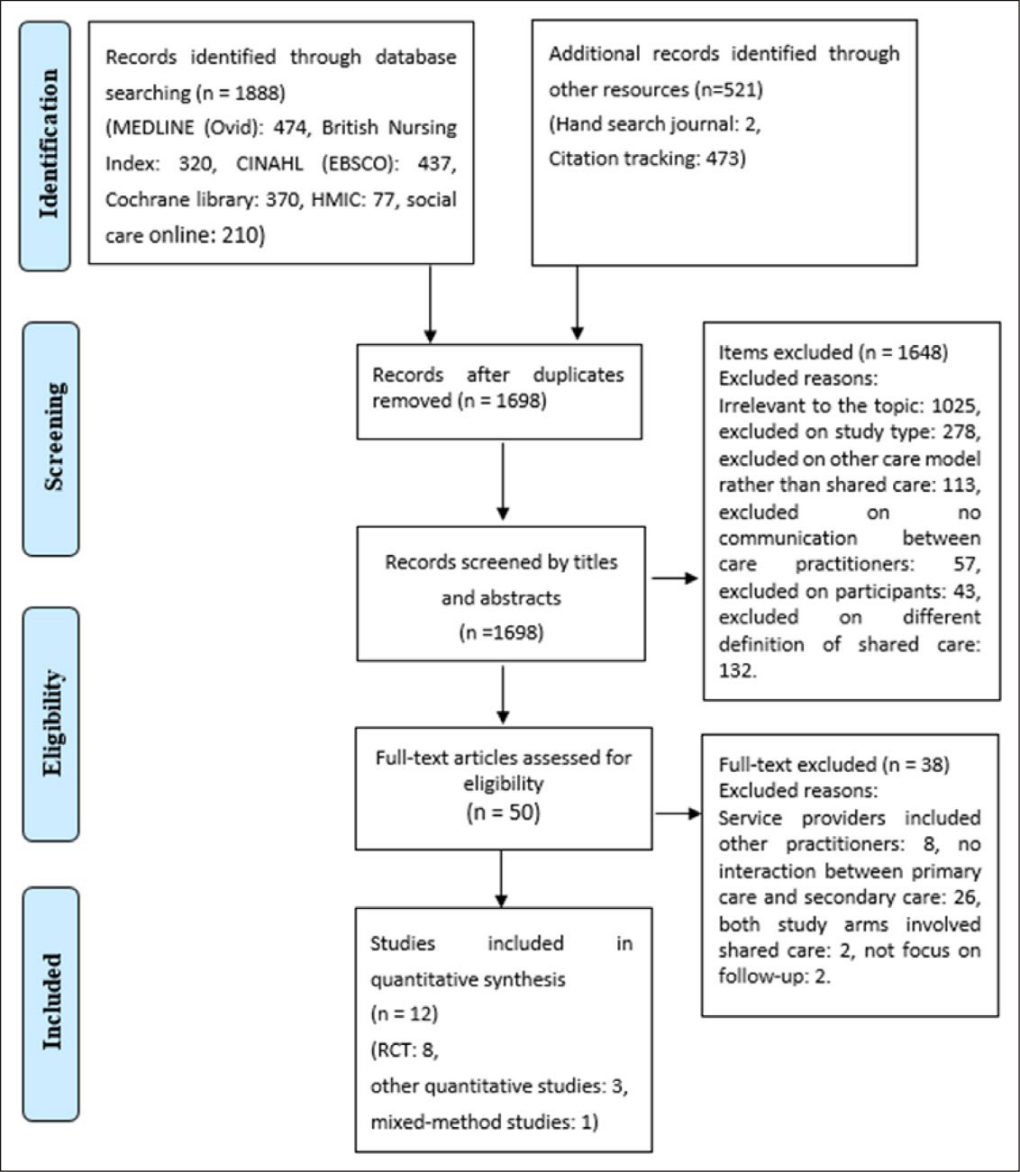
PRISMA flow diagram.

A total of 1,888 records were identified through six databases, with 521 records identified through hand searching two oncology journals and citation tracking. All records were imported into Endnote, and 1,698 records were adopted after removing the duplicates by Endnote and scanning the title and authors. Then, the remaining 1,698 records were reviewed according to the eligibility criteria by two steps. First, the title and abstract of the records were reviewed in Endnote. Next, the potential studies were downloaded, and the full-text reviewed. Finally, twelve studies met the inclusion criteria, eight of which were RCTs, three of which were observational quantitative studies, and one was a mixed-method study. The selection process is illustrated in Figure 1. The study description based on the PICO framework is illustrated in Tables 2 and 3. Besides, the essence of shared care that includes the methods of communication, the major care provider, and the length of follow-up is described in Table 4.

**Table 2:** Overview of the included randomised controlled studies.

Author, year, country	Sample and setting	Intervention group	Control group	Length of follow-up, inclusion time	Cancer type	Outcomes	Conclusion

Bergholdt, et al. [31,32], 2012, 2013, Denmark	955 newly diagnosed cancer patients (≧18 years) treated in a public regional hospital from May 2008 to February 2009 and 323 Danish general practices (GP) were enrolled to the trial.	n = 486, two nurses with oncological experience invited the patients to join an interview about rehabilitation needs, and patients were suggested to consult their GP when necessary.	n = 469, usual care	months, all patients were diagnosed as cancer within the previous 3 months	All cancers except carcinoma in situ and non- melanoma skin cancers	No significant differences between two groups with EORTC QLQ-C30 survey at 6 months or 14 months, and POMS at 14 months. No significant differences between two groups with the number of GP proactive contact nor patients’ participation in rehabilitation activities.	The multimodal intervention could not benefit patients with health-related quality of life or psychological distress. It could not improve GP proactive contact nor patients’ participation in rehabilitation activities, neither.
Nielsen, et al. [33], 2003, Denmark	248 cancer patients (≧18 years) from the oncology department of a university hospital between August 1998 and December 1998.	n = 127, the intervention includes three elements: provide GP and patients with standardised knowledge package, build the communication channels between hospital and GP, and encourage patients to contact their GPs when they had healthcare problems.	n = 121, normal procedure	6 months, both primary cancer and recurrent cancer patients were included	All types of cancer except lymphoma	Patients’ attitudes toward the shared care and their GPs is more positive in the intervention group at 3 months in 2 of 4 items (p < 0.05), and patients contacted with their GPs more than the control group (p < 0.05). However, no significant differences were detected in quality of life or performance status between two groups. The subgroup analyses indicated that men and young patients (18–49 years) tent to rate the shared care more positively.	Shared care could enhance the involvement of primary care and patients, which influenced the patients’ attitude toward healthcare services positively, especially for men and young patients.
Holtedahl, et al. [34], 2005, Norway	91 adult cancer patients (≧18 years) in a university hospital were enrolled from October 1999 to September 2000.	n = 41, two 30 minutes visits were conducted by GP and physician separately. The topic is about patients’ experiences related to cancer. Besides, patients were encouraged to contact their GPs if they had any health-related problem.	n = 50, not mentioned about the care content.	6 months, both primary cancer and recurrent cancer patients who had finished cancer therapy were included	All types of cancer except pre- cancerous conditions such as in situ cervical cancer	There was no significant difference between two arms in quality of life, patients’ satisfaction, or the consultation times with GP at six months. The relatives’ satisfaction improved in the intervention group at six months (P = 0.018).	Some patients might benefit from the GP follow-up, but the improvements were not identified this study. Besides, the intervention did not generate closer contact between patients and their GPs.
Grunfeld, et al. [35], 2006, Canada	968 early-stage breast cancer patients from six cancer centres were enrolled from January 1997 to June 2001.	n = 483, the family physician received a one-page guideline from the cancer centre. The guideline included follow-up arrangement, referral instruction, and disease relevant information. The patients received follow-up care from their family physician.	n = 485, the patients received follow-up care from cancer centre	From less than 2 years to 5 years, patients who had been diagnosed with cancer from 9 to 15 months ago and had completed treatment 3 months ago	early stage breast cancer	The percentage of recurrence, death, and serious clinical event in shared care group were 11.2%, 6.0%, and 3.5%, respectively, compared to 13.2%, 6.2%, and 3.7%, respectively, in control group. The health-related quality of life that assessed by SF-36 and HADS between two arms did not show significant differences.	The shared care follow-up did not increase the risk of important recurrence-related serious clinical event or health-related quality of life for breast cancer patients.
Blaauwbroek, et al. [36], 2008, Netherlands (historical control design)	121 adult survivors (≧18 years) who used to be diagnosed as childhood cancer in a paediatric oncology department and did not join any follow-up study agreed to enter the study in 2004 and 2005.	n = 121, three visits were carried out during three years period. Visit 1 and visit 3 were conducted by an on-site family doctor at the medical centre, visit 2 was conducted by the local family doctor. Assessments, survey, and individualised follow-up suggestion were provided in these visits.	The data from another matched study in the Netherlands was used as control group data.	years, all patients had been treated in the hospital at least 5 years ago	All childhood cancers and Langerhans-cell histiocytosis, except central nervous system tumours	At visit 1, patients in intervention arm showed lower level of health-related quality of life with RAND-36 survey compared to control group data, but no significant differences were detected at visit 3. Patient satisfaction was assessed at visit 2, and 89 (88%) of the 101 patients were satisfied with shared care model.	Shared care involved paediatric oncologists and family doctors can be used in the long-term follow-up in childhood cancer patients.
Emery, et al. [37], 2017, Australia	prostate cancer patients with low risk or moderate risk features were recruited from two rural and four urban medical centres from November 2011 to July 2013.	n = 45, a GP visit was conducted to re-engage their relationship with patients. Besides, the treatment centre provided a SCP to the patients and their GPs, and a register and recall system was built to enhance GPs’ and patients’ compliance. After that, GP replaced two hospital routine follow-up visits at 6 months and 9 months.	n = 43, usual care (the patients visited the hospital specialists every three months)	months, all patients had completed curative intent treatments within the previous 8 weeks	Prostate cancer with low risk or moderate risk features, expect the metastatic disease	No significant differences between two groups with the HADS, the CaSUN, the EPIC, and the PSQ-18 results. But the intervention group had a preference of shared care than control group (P < 0.001), and shared care costs less than usual care.	Shared care model could not benefit prostate cancer patients with distress, unmet needs, cancer-specific quality of life, or satisfaction, but it is feasible as to provide a similar outcome with less money.
Mayer, et al., [38], 2016, United States	37 cancer patients (≧21 years) who had completed curative intent treatment in a cancer hospital were recruited.	n = 20, the research nurse in the hospital made a draft SCP, then the oncology nurse practitioner made a 40 minutes transition visit with the patient and revised the SCP. The final SCP was sent to both patients and their PCPs. Besides, the PCPs received relevant knowledge about follow-up. Afterwards, the PCPs carried out a semi-structured follow-up visit with patients within four weeks.	n = 17, the same with intervention group but without GP visit	6 weeks, all patients had completed curative intent treatments within the previous 4 to 6 weeks	All types of cancer except metastatic cancer	All patients reported having less contradictory information about care (P < 0.0001) and expected less follow-up from oncologists (P = 0.03) at 6 weeks. All PCPs felt more confident (P = 0.01) about survivorship care. However, the PCP visit has little effect on the results, except making a borderline difference with worry level (P = 0.05).	The SCP could improve patients’ and PCP’s confidence in survivorship information, but no significant benefit was identified about GP visit. This is a pilot study, a larger study should explore the changes in the future.

GP = general practitioner. EORTC QLQ-C30 = the European Organisation for Research and Treatment of Cancer Quality of Life Questionnaire Core 30. POMS = Profile of Mood States Scale. RAND-36 = RAND 36-Item Health Survey. SCP: survivorship care plan. HADS = 14-item Hospital Anxiety and Depression Scale. CaSUN = Unmet needs were assessed with the Cancer Survivors’ Unmet Needs measure. EPIC = Expanded Prostate Cancer Index Composite. PSQ-18 = 18-item Short-form Patient Satisfaction Questionnaire. SF-36 = Medical Outcomes Study Short Form 36-Item General Health Survey.

**Table 3:** Overview of the other studies.

Author, year, country	Research design	Sample and setting	Procedures (Intervention and comparison)	Length of follow-up, onset time	Cancer type	Outcomes	Conclusion

Hanan, et al., [29], 2014, Ireland	Descriptive, single-centre, mixed methods	The community nurse took the responsibility of management of cancer patients instead of hospital nurses, and the visit was conducted at patients’ home.	The research included a six months skills training (from specialist cancer staff) for community nurses, specific referral form, and provided hospital support for community nurses in urgent situation by phone. The patients were told that the medical oncologists still took the responsibility of them. The patients were majorly managed by community nurses.	5 years, not mentioned	Not mentioned	Quantitative outcomes: Community nurse-led cancer care was compared with hospital activity data before the programme (baseline), in the middle of the programme (after the six months training), and five years after the baseline. The community nurses delivered safe cancer care, and the hospital attendances was decreased. The community nurses had more confidence in cancer management. Qualitative outcomes: The patients reported a shorter travel to health care, and improved quality of life. They had confidence in community nurses, and they indicated their sense of autonomy increased.	Shared care approach with training community nurses could benefit both patients and health care providers.
Blaauwbroek, et al. [39], 2012, Netherlands	Descriptive, single-centre, quantitative	80 childhood cancer survivors (≧18 years) who used to be diagnosed in a long-term follow-up clinic and did not join any follow-up study and their family doctors (n = 79) joined this study from September 2008.	The research team (hospital specialists) constructed a personalised SCP and sent the printed booklet to the patients. The plan was also accessible through a secure website to the survivors and their family doctors. Besides, the research team asked the survivors to make a half-hour meeting with their family doctors, they would remind the survivors again if they did not meet their family doctors six months later. Follow-up was done primarily by the family doctor.	one year, at least 5 years off-treatment	All types of cancer expect central nervous system tumours, survivors of all three levels of risk were included (low-risk, medium-risk, and high risk)	The family doctors were asked to finish an information form about the screening and the additional test results. Besides, an 18-item questionnaire and a 14-item questionnaire were used to assess survivors’ and family doctors’ views, respectively, about the website and the provided information, the late effects were also collected by the family doctors. Outcomes: 73 survivors and 72 family doctors completed the study. 97% of the survivors thought the follow-up was beneficial, but 11% of the survivors felt that the information was inadequate. 82% of the survivors believed in their family doctors’ ability in the follow-up. However, 77% of survivors felt worried about the reawakened memories about the disease. 60 family doctors (83%) finished all the recommended tests. 95% of the family doctors felt the follow-up was more beneficial and 83% felt their knowledge of late effects increased. Besides, 93% of the family doctors felt confident about management of follow-up if they received the SCP.	The shared care with web-based SCP is available in the long-term cancer follow-up of adults with childhood cancer, and most survivors and family doctors were satisfied with this model. Besides, both survivors and the family doctors thought their relevant knowledge improved though this process. The negative effect was the follow-up might reawaken survivors of bad memories about disease.
Berger, et al., [40], 2017, France	Descriptive, single-centre, quantitative	150 childhood cancer survivors (≧18 years) and their GPs (n = 106) involved in this study from December 2010 to June 2013. These survivors were from a region cancer registry and diagnosed of cancer before 15 years old.	The paediatric oncologist and the internist invited the survivors to join a consultation, in which the practitioners explained the medical history and potential late effects to the survivors. Additional tests were conducted based on the consultation. Besides, the medical doctor provided the consultation summaries and recommendations about follow-up (recommendation card) for the survivors and their GPs. The medical doctor would call the GPs if they did not respond to the study at first.	One year, diagnosed as primary cancer from January 1987 to December 1992.	All types of cancer expect leukaemia	120 survivors finished the nine-question satisfaction form, 107 of them (89%) were satisfied with the consultation. 86% of the survivors found the recommendation card was useful. More than 75% felt their lifestyle changed such as physical activity and diets. Type of cancer and treatment would influence the satisfaction with the follow-up (p < 0.05). The survivors who received chemotherapy felt more satisfaction with the shared care (p = 0.03). 106 GPs finished the five areas satisfaction survey, 63 of them (59%) reported they were not provided enough information about the patient’s treatment, 82 of them (77%) reported lack of late effect of chemotherapy. But most of them felt the recommendation card was useful (61%), over 80% of them appreciate the collaboration and availability of contact with hospital.	The long-term follow-up by collaboration of GP and hospital specialist was feasible and could benefit childhood cancer survivors and family physicians.
Lund, et al. [41], 2016, Denmark	Descriptive, multi-centre, quantitative	530 cancer patients from three hospitals were transferred to their GPs and invited to join the shared care follow up between September 2011 and March 2012, the follow-up lasts for three years.	The urological specialists developed follow-up recommendations for evaluating patient and GP compliance, including the schedule of follow-up and the instructions of referral. The patients were transferred to their GPs.	3 years, with or without relapse	Prostate cancer	426 (80.8%) patients were analysed, 390 patients (91.5%) were rated as “acceptable” or “good” compliance (the compliance was rated as four columns: unknown, poor, acceptable, and good); a total of 393 GP (92.3%) were rated as “good” or “acceptable” compliance (the compliance was rated as four columns: unknown, poor, acceptable, and good).	Shared care model is a safe approach with high rate of patient and GP compliance

GP = general practitioner. SCP: survivorship care plan.

**Table 4:** Essence of shared care.

Author, year, country	Major follow-up care provider in shared care	Other follow-up care provider in shared care	Method of shared care involvement	Number of formal interaction between different care providers, communication tool between care providers	Number/frequency of interaction between patients and primary care in intervention group	Number/frequency of interaction between patients and primary care in control group	Difference between two groups

Hanan, et al. [29], 2014, Ireland	Community nurses	Oncology day-ward nurses, specialist cancer staff	The shared care included a six months skills training (from specialist cancer staff) for community nurses, specific referral form, and provided hospital support for community nurses in urgent situation by phone. The patients were told that the medical oncologists still took the responsibility of them.	More than once, skill training, phone, and resource book	Not mentioned	Not mentioned	Not mentioned
Bergholdt, et al. [31,32], 2012, 2013, Denmark	General practitioner	Two hospital nurses with oncological experience	The hospital nurses suggested patients to consult their GPs when necessary. Equally, they also encouraged the GPs to be proactive to offer support to their patients, and send the GP an email, which include the patients’ information.	Once, Email and phone	Patients reported: 168 contacts (101 GP proactive contact, 61.1%)GP reported:379 contacts (232 GP proactive contact, 61.2%)	Patients reported: 156 contacts (81 GP proactive contact, 51.9%) GP reported: 373 contacts (206 GP proactive contact, 55.2%)	No significant differences in both patients reported and GP reported contacts.
Nielsen, et al. [33], 2003, Denmark	Both hospital specialists and GPs	/	Transferring knowledge and information from hospital to GP, building communication channels, encouraging patients to contact their GPs	Once, ordinary mail	Not mentioned	Not mentioned	More patients had contact with their GP in intervention group (p = 0.049 at 3 months, p = 0.046 at 6 months)
Holtedahl, et al. [34], 2005, Norway	Both GP and hospital physician	/	The GP was asked to initiate a consultation with patients. The patients were encouraged to contact their GPs if they have health related problem.	Once, unclear	The average consultation time was 1.26 per patients	The average consultation time was 1.04 per patients	No significant difference between two arms
Grunfeld, et al. [35], 2006, Canada	Family physician	Practitioner in cancer centre	The family physician received a one-page guideline from the cancer centre. The guideline included follow-up arrangement, referral instruction, and disease relevant information.	Once, unclear	Not mentioned	Not mentioned	Not mentioned
Blaauwbroek, et al. [36], 2008, Netherlands	Both paediatric oncologists and family doctors	/	The medical centre advised the patients to meet their family doctors. The patient information and the test results were shared by medical centre and primary care.	More than once, email or telephone	Not mentioned	Not mentioned	Not mentioned
Emery, et al. [37], 2017, Australia	Both hospital specialists and GPs	/	The treatment centre faxed a SCP to the GP. The register and recall system sent GP follow-up reminder letters.	More than once, fax, letter	Not mentioned	Not mentioned	Not mentioned
Mayer, et al. [38], 2016, United states	Both hospital nurses and GPs	/	Hospital nurses provided SCP to both patients and PCPs, they also conducted a transition visit with the survivors. The PCP carried out a semi-structured follow-up visit with survivors (talking points were developed by the hospital).	More than once, Electronic health system, mail, or email, websites	Not mentioned	Not mentioned	Not mentioned
Blaauwbroek, et al. [39], 2012, Netherlands	Family doctors	Long-term follow-up clinic	The research team (hospital specialists) constructed a personalised SCP website which was accessible to the family doctors. Besides, the research team asked the survivors to make a half-hour meeting with their family doctors, they reminded the survivor again if they did not meet their family doctors six months later.	More than once, secure website or letter	Not mentioned	Not mentioned	Not mentioned
Berger, et al. [40], 2017, France	GP	A paediatric oncologist and an internist	The medical doctor provided the consultation summaries and recommendations about follow-up for the survivors and their GPs. The medical doctor would call the GPs if they did not respond to the study at first.	Once, unclear	Not mentioned	Not mentioned	Not mentioned
Lund, et al. [41], 2016, Denmark	GP	Hospital outpatient urological specialists	The urological specialists developed follow-up recommendations for evaluating patient and GP compliance, including the schedule of follow-up and the instructions of referral.	Once, unclear	Not mentioned	Not mentioned	Not mentioned

GP = general practitioner. SCP: survivorship care plan.

### Data extraction and quality appraisal

All studies except the mixed-method study [29] were considered as excellent or good quality, and were included. To keep a balance between having confidence about the findings by only including good quality evidence and comprising enough evidence in order to answer the research question, only those studies which were considered as bad quality were excluded [30]. As a result, this review finally includes seven RCTs reported in eight papers and three descriptive quantitative studies. One RCT was reported in two papers which assessed different types of outcomes [31,32]. The results of the quality appraisal of the studies can be found in Tables 5 and 6 (included studies), and Appendix 7 (excluded study), and the examples of the appraisal process with the checklist can be found in Appendix 5, 6, and 7.

**Table 5:** Critical review of the RCTs with Critical Appraisal Skills Programme (CASP).

Section/Question	Score							

	Bergholdt, et al., 2012 [31]	Bergholdt, et al., 2013 [32]	Nielsen, et al., 2003 [33]	Holtedahl, et al., 2005 [34]	Grunfeld, et al., 2006 [35]	Blaauwbroek, et al., 2008 [36]	Emery, et al., 2017 [37]	Mayer, et al., 2016 [38]

(A) Are the results of the trial valid?								
1. Did the trial address a clearly focused issue?	2	2	2	1	2	2	2	1
2. Was the assignment of patients to treatments randomised?	2	2	2	2	2	2	2	2
3. Were patients, health workers and study personnel blinded?	0	0	0	0	0	0	0	1
4. Were the groups similar at the start of the trial?	2	2	1	1	2	1	2	2
5. Aside from the experimental intervention, were the groups treated equally?	1	1	1	1	2	0	1	2
6. Were all of the patients who entered the trial properly accounted for at its conclusion?	2	2	2	2	2	2	2	2
(B) What are the results?								
7. How large was the treatment effect?	2	2	2	1	2	2	2	1
8. How precise was the estimate of the treatment effect?	2	2	2	2	1	2	2	1
(C) Will the results help locally?								
9. Can the results be applied in your context? (or to the local population?)	1	1	2	1	0	1	1	1
10. Were all clinically important outcomes considered?	1	2	2	1	2	2	1	1
11. Are the benefits worth the harms and costs?	2	2	2	2	2	2	2	2
Total score (maximum 22)	17	18	18	14	17	16	17	16

“0” represents many limitations, “1” represents some limitation, “2” represents excellent.

**Table 6:** Critical review of the quantitative studies with Health Care Practice R&D Unit (HCPRDU).

Question	Score		

	Blaauwbroek, et al., 2012 [39]	Berger, et al., 2017 [40]	Lund, et al., 2016 [41]

(1) Study overview	2	2	1
(2) Study, setting, sample and ethics	2	2	1
(3) Ethics	2	0	1
(4) Group comparability and outcome measurement	1	1	1
(5) Policy and practice implications	1	1	2
(6) Other comments	2	1	2
Total score (maximum 12)	10	7	8

“0” represents many limitations, “1” represents some limitation, “2” represents excellent.

### Effectiveness of shared care

#### Physical and psychological health status

A total of seven papers assessed whether shared care could improve the survivors’ health status, including both physical and psychological improvements. Among those six papers that detected the quality of life, five papers detected the differences between the two arms at the end of the intervention, and one paper compared the outcomes between the beginning and the end of the shared care, but none of these papers detected any significant difference [31,33,34,35,36,37]. As for the survivors’ psychological status, the two papers evaluated the survivors’ psychological distress level, finding no significant difference between the intervention group and control group [31,37]. Besides, the paper that assessed the survivorship worries found no difference before and after the shared care, but there was a borderline difference between the two groups after the shared care [38]. Turning to the survivors’ physical conditions, the paper that evaluated the survivors’ performance status found no significant differences between the two arms [33]. Further, the study used a non-inferiority design to evaluate the number of recurrences, death, and the serious clinical event could not demonstrate whether the intervention group was worse than the control group [35].

#### Satisfaction, attitudes and needs towards health care

Seven out of eleven papers evaluated the survivors’ attitudes and needs toward the shared care. The results include the satisfaction with the follow-up [34,37,38], the satisfaction towards the PCPs [36], the attitudes toward the cooperation between health providers [33], the attitudes toward the information they received [39], survivorship unmet needs [37], and the preference for the care provider [37,38]. From the survivor report results, the cooperation between health care practitioners improved (p = 0.025 and p = 0.004 in two out of four items) in the intermediate outcomes [33], and Emery et al. found the survivors in the shared care group would prefer shared care in the follow-up after the study (p = 0.07) [37]. Apart from these, the studies found no other significant differences between the two arms.

Some studies used a descriptive way to assess the satisfaction of survivors and got similar results. First, Blaauwbroek et al. found that 88% of the survivors who completed the questionnaire were satisfied with the follow-up [36], while Berger et al. found a similar result in that 89% of the survivors who finished the questionnaire were satisfied with the health care [40]. Second, more than 80% of the survivors were generally satisfied with the information they received in the follow-up in two studies, and 71% of the survivors followed the instructions [39,40]. Third, Blaauwbroek et al. reported that survivors were more aware of the benefits of follow-up (90.2%), and 73.6% of the survivors were more confident with the GPs’ capacity [39]. The only disadvantage of shard care reported in all eleven studies was that 15.3% of the childhood cancer survivors mentioned that the information they received reminded them of the negative memories from the past [39]. Finally, the subgroup analysis found that the men and younger age group (18–49 years) were significantly more satisfied with the shared care and found it easier to accept the GPs as their care provider [33], and the diagnosis and the treatment could affect the satisfaction with the follow-up (p < 0.05) [40].

#### Care referral and continuity of care

The studies evaluated the continuity of care in different aspects, such as the primary care practitioner’s confidence in survivorship knowledge, their attitudes toward the follow-up and the information they received during the follow-up, and the frequency that survivors participated in the follow-up, but no significant differences were found between the intervention group and control group [32,38]. Besides, most studies used descriptive data to report the outcomes. Blaauwbroek et al. found that 71.7%–77.4% of family doctors reported that their knowledge and ability of providing follow-up care were improved [39], and Lund et al. reported that 91.5%–92.3% could follow the follow-up recommendations [41]. Besides, Blaauwbroek et al. found that 82% of the family doctors were satisfied with the cooperation and the information they received [36]. Another study reported that 61% of GPs considered the information they received and 82% ranked the collaboration with hospital as helpful, 59%–77% of the general practitioners stated that they received insufficient information in different aspects. Furthermore, GPs recommended that specific cancers needed particular follow-up more than other cancers such as more GPs considering that renal tumour survivors needed more specific care than lymphomas survivors (p = 0.013) [40].

#### The cost of shared care

Only one paper compared the cost of shared care with usual care and found that shared care was cheaper than usual care [37]. In the Emery et al. study, a multisite randomised controlled trial which included patients of two rural and four urban treatment centres was conducted. Five routine follow-up visits were carried out in both two groups, and two hospital visits were replaced by GP in the experimental group. At the end of the research, the shared care spent $323 less than usual care for each patient in the one-year follow-up.

### The care in the shared care group and control group

The interventions were complex. Three trials implemented the shared care with a clear division of tasks by hospital specialists and primary care physicians [34,36,37], while the other seven trials implemented the shared care by intending to transfer the follow-up care to primary care providers smoothly with specified information exchange [31,32,33,35,38,39,40,41]. The hospital centre and primary care formally communicated with each other more than once in four out of eleven trials [36,38,39], but the others only involved one formal communication [31,32,33,34,35,40,41]. The cancer survivors in the control group were followed up by usual care [31,32,33,37] or in the hospital [35] in most of the trials. However, one study [38] did not include survivors in the control group but applied the data from another matched study in the same country as the control group. Besides, two studies did not clearly describe the content of the control group [34,38].

## Discussion

This review included 11 papers that evaluated shared care in the continuity of care for cancer survivors. These studies conducted shared care with various and complex multifaceted interventions for improving the follow-up of cancer survivors, especially their quality of life and depression. An overview of the results in the selected studies suggests that survivors and general practitioners reported favouring shared care, and the survivors who had experienced shared care had a stronger preference for shared care in the future. However, there were no significant differences in terms of quality of life, mental health outcomes, unmet needs, and serious clinical events between shared care and usual care. One important confounding factor might be that the patient-reported results could have been affected by the lack of confidence in primary care [42] since it is impossible to blind the survivors.

Although only 11 papers were included in the present review, the overall sample size and the quality of studies constitutes an overview with a preliminary picture of the possible way to conduct shared care and the effectiveness of shared care. Two models of shared care were identified as offering potential to improve the monitoring of cancer survivors: the transference of survivors, which lies within the information exchange; and the coordination of assessments and treatments, which allows distant health professionals to conduct the monitoring alternately. Several interventional strategies that were utilised played a role in enhancing the efforts in terms of care cooperation: (1) survivorship care plan; (2) referral and consultation visit; (3) improving the knowledge of PCPs; (4) enhancing patient’s confidence in health care practitioners, especially in PCPs; (5) building the communication channel between health care professionals; and (6) the register and recall system.

The studies that assessed the continuity of care found that shared care could meet the requirements of follow-up, and the PCPs felt their knowledge was improved and that they had the capability of providing healthcare with the support of hospital specialists. Blaauwbroek et al. found that 77.4% of the PCPs considered that they had the capacity of providing follow-up if the SCP was available [39], while another survey found that only 40% of the PCPs felt confident of their knowledge in the follow-up of cancer survivors in the usual care [43]. Besides, the only study that compared the cost of shared care to usual care found that the shared care on average reduced costs by $323 per patient at one-year follow-up [37].

Although only those studies that were rated as “good” or “excellent” were included in this review, several studies had major limitations, such as the sample size being insufficient [34,38], the significant differences at the baseline [33], and the outcome assessor not being blinded in most of the trials. Besides, there were only six RCTs that compared shared care with usual care, and many of the results were illustrated within a descriptive method. Thus, the small number of available studies could not provide a solid foundation for this review. Furthermore, there were various types of outcomes that were detected in the studies, so many of the results could not be regrouped based on the considerable heterogeneity. Besides, all the studies were conducted in developed countries, and most of the studies were performed in city settings, so the results might not apply to other undeveloped countries or rural regions. Further limitations include that only papers written in English were included and the author appraised the papers without blinding to the published journal or the writers.

## Conclusions

### Implications for practice

The present review shines a light on improving the follow-up, with current evidence indicating that shared care is an affordable model as well as being feasible and acceptable for cancer survivors. It enables GP’s involvement in survivorship care and help the cooperation between hospital and primary care. Although the evidence showed that the effectiveness of shared care is similar to hospital follow-up, the strategies we identified from the included studies could be useful to all stakeholders of health care and provide a preference for implementing new strategies in cancer follow-up to address the sustainable burden of hospitals. Due to limited evidence of financial analysis, we could not make conclusion that shared care is cheaper than usual care, but it is potentially contributing to help resolve the stabilised health care costs. However, more solid evidence about the effectiveness of shared care is needed before it can be routinely implemented.

### Implications for research

Although the results of this review do not confirm that shared care is more effective than usual care in the management of follow-up in cancer survivors, several key elements have been identified in shared care: the consultation meeting, the formal transferring of documents, encouraging communication between the survivors and the practitioners, and the length of follow-up. Besides, the communication channel and register and recall system are also considered as important elements in shared care. The research gaps of the included studies also indicate the directions that future studies need to address. First, further RCTs with sufficient sample size are needed to explore the health-related cost of shared care and the clinical outcomes. Second, the differences in the subgroup indicate that individual follow-up should be conducted based on the diagnosis, treatment, age, and gender. Third, the follow up should be modified according to the specific health care needs in different time frames since diagnosis [44].

## Additional Files

The additional files for this article can be found as follows:

10.5334/ijic.3954.s1 Appendix 1 MEDLINE (Ovid) and HMIC search strategy.

10.5334/ijic.3954.s1 Appendix 2 Critical Appraisal Skills Programme (CASP) Randomised Controlled Trials Checklist.

10.5334/ijic.3954.s1 Appendix 3 Health Care Practice R&D Unit (HCPRDU) quantitative research checklists.

10.5334/ijic.3954.s1 Appendix 4 Health Care Practice R&D Unit (HCPRDU) mixed methods research checklists.

10.5334/ijic.3954.s1 Appendix 5 Example of using CASP RCT checklist to appraise a selected RCT.

10.5334/ijic.3954.s1 Appendix 6 Example of using HCPRDU quantitative research checklists to appraise a selected quantitative study.

10.5334/ijic.3954.s1 Appendix 7 Example of using HCPRDU mixed methods research checklists to appraise a selected mixed methods’ study.
