# Supplementary material for: The Effectiveness of Shared Care in Cancer Survivors—A Systematic Review
Source: Int J Integr Care. 2018 Oct 12;18(4):2. doi: 10.5334/ijic.3954 (PMC6199565; doi:10.5334/ijic.3954)
Supplement: Appendix 1 — MEDLINE (Ovid) and HMIC search strategy. [file ijic-18-4-3954-s1.pdf]

Appendice 1

MEDLINE (Ovid) and HMIC search strategy

1. cancer.mp. or Neoplasms/

2. cancer*.mp. [mp=title, abstract, original title, name of substance word, subject heading word, keyword heading word, protocol supplementary concept word, rare disease supplementary concept word, unique identifier, synonyms]

3. neoplas*.mp. [mp=title, abstract, original title, name of substance word, subject heading word, keyword heading word, protocol supplementary concept word, rare disease supplementary concept word, unique identifier, synonyms]

4. malignan*.mp. [mp=title, abstract, original title, name of substance word, subject heading word, keyword heading word, protocol supplementary concept word, rare disease supplementary concept word, unique identifier, synonyms]

5. carcinoma*.mp. [mp=title, abstract, original title, name of substance word, subject heading word, keyword heading word, protocol supplementary concept word, rare disease supplementary concept word, unique identifier, synonyms]

6. sarcoma*.mp. [mp=title, abstract, original title, name of substance word, subject heading word, keyword heading word, protocol supplementary concept word, rare disease supplementary concept word, unique identifier, synonyms]

7. oncolog*.mp. [mp=title, abstract, original title, name of substance word, subject heading word, keyword heading word, protocol supplementary concept word, rare disease supplementary concept word, unique identifier, synonyms]

8. tumo?r*.mp. [mp=title, abstract, original title, name of substance word, subject heading word, keyword heading word, protocol supplementary concept word, rare disease supplementary concept word, unique identifier, synonyms]

9. adenocarcinoma*.mp. [mp=title, abstract, original title, name of substance

word, subject heading word, keyword heading word, protocol supplementary concept word, rare disease supplementary concept word, unique identifier, synonyms]

10. infiltrat*.mp. [mp=title, abstract, original title, name of substance word, subject heading word, keyword heading word, protocol supplementary concept word, rare disease supplementary concept word, unique identifier, synonyms]

11. medullary.mp. [mp=title, abstract, original title, name of substance word, subject heading word, keyword heading word, protocol supplementary concept word, rare disease supplementary concept word, unique identifier, synonyms]

12. intraductal.mp. [mp=title, abstract, original title, name of substance word, subject heading word, keyword heading word, protocol supplementary concept word, rare disease supplementary concept word, unique identifier, synonyms]

13. 1 or 2 or 3 or 4 or 5 or 6 or 7 or 8 or 9 or 10 or 11 or 12

14. follow-up.mp. [mp=title, abstract, original title, name of substance word, subject heading word, keyword heading word, protocol supplementary concept word, rare disease supplementary concept word, unique identifier, synonyms]

15. followup*.mp. [mp=title, abstract, original title, name of substance word, subject heading word, keyword heading word, protocol supplementary concept word, rare disease supplementary concept word, unique identifier, synonyms]

16. follow-up care.mp. [mp=title, abstract, original title, name of substance word, subject heading word, keyword heading word, protocol supplementary concept word, rare disease supplementary concept word, unique identifier, synonyms]

17. follow-up stud*.mp. [mp=title, abstract, original title, name of substance word, subject heading word, keyword heading word, protocol supplementary concept word, rare disease supplementary concept word, unique identifier, synonyms]

18. postsurgery.mp. [mp=title, abstract, original title, name of substance word,

subject heading word, keyword heading word, protocol supplementary concept word, rare disease supplementary concept word, unique identifier, synonyms]

19. post surgery.mp. [mp=title, abstract, original title, name of substance word, subject heading word, keyword heading word, protocol supplementary concept word, rare disease supplementary concept word, unique identifier, synonyms]

20. postsurgical*.mp. [mp=title, abstract, original title, name of substance word, subject heading word, keyword heading word, protocol supplementary concept word, rare disease supplementary concept word, unique identifier, synonyms]

21. post surgical*.mp. [mp=title, abstract, original title, name of substance word, subject heading word, keyword heading word, protocol supplementary concept word, rare disease supplementary concept word, unique identifier, synonyms]

22. postoperat*.mp. [mp=title, abstract, original title, name of substance word, subject heading word, keyword heading word, protocol supplementary concept word, rare disease supplementary concept word, unique identifier, synonyms]

23. post-operat*.mp. [mp=title, abstract, original title, name of substance word, subject heading word, keyword heading word, protocol supplementary concept word, rare disease supplementary concept word, unique identifier, synonyms]

24. continuity of patient care.mp. [mp=title, abstract, original title, name of substance word, subject heading word, keyword heading word, protocol supplementary concept word, rare disease supplementary concept word, unique identifier, synonyms]

25. disease management.mp. [mp=title, abstract, original title, name of substance word, subject heading word, keyword heading word, protocol supplementary concept word, rare disease supplementary concept word, unique identifier, synonyms]

26. surveillance.mp. [mp=title, abstract, original title, name of substance word, subject heading word, keyword heading word, protocol supplementary concept word, rare disease supplementary concept word, unique identifier,

synonyms]

27. routine test*.mp. [mp=title, abstract, original title, name of substance word, subject heading word, keyword heading word, protocol supplementary concept word, rare disease supplementary concept word, unique identifier, synonyms]

28. disease progression.mp. [mp=title, abstract, original title, name of substance word, subject heading word, keyword heading word, protocol supplementary concept word, rare disease supplementary concept word, unique identifier, synonyms]

29. 14 or 15 or 16 or 17 or 18 or 19 or 20 or 21 or 22 or 23 or 24 or 25 or 26 or 27 or 28

30. Patient Care Team/ or Primary Health Care/ or Family Practice/

31. shared care.mp. [mp=title, abstract, original title, name of substance word, subject heading word, keyword heading word, protocol supplementary concept word, rare disease supplementary concept word, unique identifier, synonyms]

32. sharing of care.mp. [mp=title, abstract, original title, name of substance word, subject heading word, keyword heading word, protocol supplementary concept word, rare disease supplementary concept word, unique identifier, synonyms]

33. co-management.mp. [mp=title, abstract, original title, name of substance word, subject heading word, keyword heading word, protocol supplementary concept word, rare disease supplementary concept word, unique identifier, synonyms]

34. collaborative care.mp. [mp=title, abstract, original title, name of substance word, subject heading word, keyword heading word, protocol supplementary concept word, rare disease supplementary concept word, unique identifier, synonyms]

35. care coordination.mp. [mp=title, abstract, original title, name of substance word, subject heading word, keyword heading word, protocol supplementary concept word, rare disease supplementary concept word, unique identifier, synonyms]

36. coordinated care.mp. [mp=title, abstract, original title, name of substance word, subject heading word, keyword heading word, protocol supplementary concept word, rare disease supplementary concept word, unique identifier, synonyms]

37. (referral and consultation).mp. [mp=title, abstract, original title, name of substance word, subject heading word, keyword heading word, protocol supplementary concept word, rare disease supplementary concept word, unique identifier, synonyms]

38. cooperative behavio?r.mp. [mp=title, abstract, original title, name of substance word, subject heading word, keyword heading word, protocol supplementary concept word, rare disease supplementary concept word, unique identifier, synonyms]

39. delivery of health care.mp. [mp=title, abstract, original title, name of substance word, subject heading word, keyword heading word, protocol supplementary concept word, rare disease supplementary concept word, unique identifier, synonyms]

40. 30 or 31 or 32 or 33 or 34 or 35 or 36 or 37 or 38 or 39

41. shared service*.mp. [mp=title, abstract, original title, name of substance word, subject heading word, keyword heading word, protocol supplementary concept word, rare disease supplementary concept word, unique identifier, synonyms]

42. 40 or 41

43. 13 and 29 and 42

44. limit 43 to (humans)

MEDLINE results: 474

HMIC results:77

British Nursing Index search strategy

(ab(follow up) OR ab(follow-up) OR ab(followup*) OR ab(postsurgery) OR ab(post-surgery) OR ab(postsurgical*) OR ab(post surgical*) OR

ab(postoperat*) OR ab(post-operat*) OR ab(continuity of patient care) OR ab(disease management) OR ab(surveillance) OR ab(routine test*) OR ab(disease progression) OR ab(aftercare) OR ab(survivorship)) AND (ab(shared care) OR ab(sharing of care) OR ab(co-management) OR ab(collaborative care) OR ab(care coordination) OR ab(coordinated care) OR ab(referral AND consultation) OR ab(cooperative behavio*r) OR ab(delivery of health care) OR ab(shared service*)) AND ((SU.EXACT("Cancer : Pain") OR SU.EXACT("Lung Cancer") OR SU.EXACT("Colorectal Cancer") OR SU.EXACT("Cervical Cancer") OR SU.EXACT("Cancer : Services") OR SU.EXACT("Cancer : Children") OR SU.EXACT("Cancer : Nursing") OR SU.EXACT("Cancer : Radiotherapy") OR SU.EXACT("Skin Cancer") OR SU.EXACT("Cancer : Chemotherapy") OR SU.EXACT("Testicular Cancer") OR SU.EXACT("Head and Neck Cancer") OR SU.EXACT("Cancer : Surgery") OR SU.EXACT("Cancer : Counselling") OR SU.EXACT("Cancer") OR SU.EXACT("Ovarian Cancer") OR SU.EXACT("Breast Cancer") OR SU.EXACT("Prostate Cancer") OR SU.EXACT("Cancer : Alternative Therapies")) OR cancer* OR neoplas* OR malignan* OR carcinoma* OR sarcoma* OR oncolog* OR tumo*r* OR adenocarcinoma* OR infiltrat* OR medullary OR intraductal)

Results: 320

CINAHL (EBSCO) search strategy

S48	S13 AND S33 AND S47	437
S47	S35 OR S36 OR S37 OR S38 OR S39 OR S40 OR S41 OR S42 OR S43 OR S44 OR S45 OR S46	24,431
S46	"inter-organizational coordination"	0
S45	"shared model"	13
S44	"integrated care"	1,496
S43	"delivery of health care"	633
S42	"shared service*"	459
S41	"cooperative behavio?r"	13

S40	"referral and consultation"	19,269
S39	"coordinated care"	419
S38	"care coordination"	1,438
S37	"collaborative care"	844
S36	"sharing of care"	66
S35	co-management	94
S34	(MM "Shared Services, Health Care") OR "shared care"	686
S33	S14 OR S15 OR S16 OR S17 OR S18 OR S19 OR S20 OR S21 OR S22 OR S23 OR S24 OR S25 OR S26 OR S27 OR S28 OR S29 OR S30 OR S31 OR S32	335,613
S32	post treatment	5,321
S31	post-treatment	3,311
S30	rehabilitation	113,235
S29	posttreatment	2,349
S28	survivorship	2,606
S27	aftercare	607
S26	"disease progression"	23,343
S25	surveillance	29,431
S24	"disease management"	10,805
S23	"continuity of patient care"	8,484
S22	post-operat*	4,089
S21	postoperat*	66,470
S20	post surgical*	1,083

S19	postsurgical*	1,715
S18	post surgery	1,704
S17	postsurgery	752
S16	followup*	3,533
S15	follow up	98,975
S14	(MM "After Care") OR "follow-up"	100,285
S13	S1 OR S2 OR S3 OR S4 OR S5 OR S6 OR S7 OR S8 OR S9 OR S10 OR S11 OR S12	286,237
S12	intraductal	424
S11	medullary	1,152
S10	infiltrat*	6,491
S9	adenocarcinoma*	7,454
S8	tumo?r*	9,360
S7	oncolog*	41,735
S6	sarcoma	4,735
S5	carcinoma*	30,047
S4	malignan*	22,717
S3	neoplas*	193,057
S2	cancer*	178,269
S1	(MM "Cancer Patients") OR (MM "Cancer Survivors") OR "cancer"	174,457

Results: 437

Cochrane library search strategy

#1 MeSH descriptor: [Neoplasms] explode all trees

#2 cancer*:ti,ab,kw (Word variations have been searched)

#3 neoplas*:ti,ab,kw (Word variations have been searched)

#4 malignan*:ti,ab,kw (Word variations have been searched)

#5 carcinoma*:ti,ab,kw (Word variations have been searched)

#6 sarcoma*:ti,ab,kw (Word variations have been searched)

#7 oncolog*:ti,ab,kw (Word variations have been searched)

#8 tumo?r*:ti,ab,kw (Word variations have been searched)

#9 adenocarcinoma*:ti,ab,kw (Word variations have been searched)

#10 infiltrat*:ti,ab,kw (Word variations have been searched)

#11 medullary:ti,ab,kw (Word variations have been searched)

#12 intraductal:ti,ab,kw (Word variations have been searched)

#13 #1 or #2 or #3 or #4 or #5 or #6 or #7 or #8 or #9 or #10 or #11 or #12

#14 MeSH descriptor: [Aftercare] explode all trees

#15 "follow up*" (Word variations have been searched)

#16 follow-up*:ti,ab,kw (Word variations have been searched)

#17 followup*:ti,ab,kw (Word variations have been searched)

#18 postsurgery:ti,ab,kw (Word variations have been searched)

#19 post surgery (Word variations have been searched)

#20 postsurgical*:ti,ab,kw (Word variations have been searched)

#21 post surgical*:ti,ab,kw (Word variations have been searched)

#22 post-surgery:ti,ab,kw (Word variations have been searched)

#23 post-surgical*:ti,ab,kw (Word variations have been searched)

#24 postoperat*:ti,ab,kw (Word variations have been searched)

#25 post-operat*:ti,ab,kw (Word variations have been searched)

#26 post operat*:ti,ab,kw (Word variations have been searched)

#27 "continuity of patient care" (Word variations have been searched)

#28 "disease management" (Word variations have been searched)

#29 surveillance:ti,ab,kw (Word variations have been searched)

#30 "disease progression" (Word variations have been searched)

#31 aftercare (Word variations have been searched)

#32 survivorship (Word variations have been searched)

#33 "post treatment" (Word variations have been searched)

#34 posttreatment (Word variations have been searched)

#35 rehabilitation (Word variations have been searched)

#36 #14 or #15 or #16 or #17 or #18 or #19 or #20 or #21 or #22 or #23 or #24 or #25 or #26 or #27 or #28 or #29 or #30 or #31 or #32 or #33 or #34 or #35

#37 MeSH descriptor: [Referral and Consultation] explode all trees

#38 shared care:ti,ab,kw (Word variations have been searched)

#39 sharing of care:ti,ab,kw (Word variations have been searched)

#40 shared service*:ti,ab,kw (Word variations have been searched)

#41 collaborative care:ti,ab,kw (Word variations have been searched)

#42 co-management (Word variations have been searched)

#43 care coordination:ti,ab,kw (Word variations have been searched)

#44 coordinated care:ti,ab,kw (Word variations have been searched)

#45 referral and consultation:ti,ab,kw (Word variations have been searched)

#46 cooperative behavio?r:ti,ab,kw (Word variations have been searched)

#47 "delivery of health care" (Word variations have been searched)

#48 "integrated care" (Word variations have been searched)

#49 "shared model" (Word variations have been searched)

#50 inter-organizational coordination (Word variations have been searched)

#51 #37 or #38 or #39 or #40 or #41 or #42 or #43 or #44 or #45 or #46 or #47 or #48 or #49 or #50 in Trials and Economic Evaluations (Word variations have been searched)

#52 #13 and #36 and #51

Results: 370

Social care online search strategy

shared care-5/7/2017:

cancer

- AllFields:'cancer*'
- OR AllFields:'neoplas*'
- OR AllFields:'malignan*'
- OR AllFields:'carcinoma*'
- OR AllFields:'sarcoma*'
- OR AllFields:'oncolog*'
- OR AllFields:'tumor*'

- OR AllFields:'adenocarcinoma*'
- OR AllFields:'infiltrat*'
- OR AllFields:'medullary'
- OR AllFields:'intraductal'

AND

follow up-5/7/2017

- AllFields:'aftercare'
- OR AllFields:'"follow up*"'
- OR AllFields:'follow-up*'
- OR AllFields:'followup*'
- OR AllFields:'postsurgery'
- OR AllFields:'post surgery'
- OR AllFields:'postsurgical*'
- OR AllFields:'post surgical*'
- OR AllFields:'post-surgery'
- OR AllFields:'post-surgical*'
- OR AllFields:'postoperat*'
- OR AllFields:'post-operat*'
- OR AllFields:'post operat*'
- OR AllFields:'"continuity of patient care"'
- OR AllFields:'"disease management"'

- OR AllFields:'surveillance'
- OR AllFields:'"disease progression"'
- OR AllFields:'survivorship'
- OR AllFields:'"post treatment"'
- OR AllFields:'posttreatment'
- OR AllFields:'post treatment'
- OR AllFields:'rehabilitation'

AND

shared care-5/7/2017

- AllFields:'shared care'
- OR AllFields:'sharing of care'
- OR AllFields:'shared service*'
- OR AllFields:'collaborative care'
- OR AllFields:'co-management'
- OR AllFields:'care coordination'
- OR AllFields:'coordinated care'
- OR AllFields:'referral and consultation'
- OR AllFields:'cooperative behavio r'
- OR AllFields:'"delivery of health care"'
- OR AllFields:'"integrated care"'
- OR AllFields:'"shared model"'

- OR AllFields:'inter-organizational coordination'
- OR AllFields:'referral and consultation'
- OR AllFields:'coordination of care'
- OR AllFields:'team-working'
- OR AllFields:'partnership*'

Results: 210

Appendice 2 Critical Appraisal Skills Programme (CASP) Randomised Controlled Trials Checklist

11 questions to help you make sense of a trial

How to use this appraisal tool

Three broad issues need to be considered when appraising the report of a randomised controlled trial:

- Are the results of the trial valid? (Section A)
- What are the results? (Section B)
- Will the results help locally? (Section C)

The 11 questions on the following pages are designed to help you think about these issues systematically.
The first two questions are screening questions and can be answered quickly. If the answer to both is *yes*, it is worth proceeding with the remaining questions.

There is some degree of overlap between the questions, you are asked to record a *yes*, *no* or *can't tell* to most of the questions. A number of prompts are given after each question. These are designed to remind you why the question is important. Record your reasons for your answers in the spaces provided.

There will not be time in the small groups to answer them all in detail!

These checklists were designed to be used as educational tools as part of a workshop

©CASP This work is licensed under the Creative Commons Attribution - NonCommercial-ShareAlike 3.0 Unported License. To view a copy of this license, visit http://creativecommons.org/licenses/by-nc-sa/3.0/. www.casp-uk.net

(A) Are the results of the trial valid?

Screening Questions

1. Did the trial address a clearly focused issue? ❑ Yes ❑ Can't tell ❑ No

Consider: An issue can be 'focused' In terms of

- The population studied
- The intervention given
- The comparator given
- The outcomes considered

---

2. Was the assignment of patients to treatments randomised? ❑ Yes ❑ Can't tell ❑ No

Consider:

- How was this carried out, some methods may produce broken allocation concealment
- Was the allocation concealed from researchers?

Is it worth continuing?

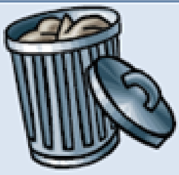

Detailed questions

3. Were patients, health workers and study personnel blinded? ☐ Yes ☐ Can't tell ☐ No

Consider:

- Health workers could be; clinicians, nurses etc
- Study personnel – especially outcome assessors

4. Were the groups similar at the start of the trial? ☐ Yes ☐ Can't tell ☐ No

Consider: Look at

- Other factors that might affect the outcome such as age, sex, social class, these may be called baseline characteristics

5. Aside from the experimental intervention, were the groups treated equally? ☐ Yes ☐ Can't tell ☐ No

6. Were all of the patients who entered the trial properly accounted for at its conclusion?

❑ Yes ❑ Can't tell ❑ No

Consider:

- Was the trial stopped early?
- Were patients analysed in the groups to which they were randomised?

(B) What are the results?

7. How large was the treatment effect?

Consider:

- What outcomes were measured?
- Is the primary outcome clearly specified?
- What results were found for each outcome?
- Is there evidence of selective reporting of outcomes?

8. How precise was the estimate of the treatment effect?

Consider:

- What are the confidence limits?
- Were they statistically significant?

(C) Will the results help locally?

9. Can the results be applied in your context? (or to the local population?)

❑ Yes ❑ Can't tell ❑ No

Consider:

- Do you have reason to believe that your population of interest is different to that in the trial
- If so, in what way?

10. Were all clinically important outcomes considered?

❑ Yes ❑ Can't tell ❑ No

Consider:

- Is there other information you would like to have seen?
- Was the need for <u>this</u> trial clearly described?

11. Are the benefits worth the harms and costs?

❑ Yes ❑ Can't tell ❑ No

Consider:

- Even if this is not addressed by the trial, what do you think?

Appendice 3 Health Care Practice R&D Unit (HCPRDU) quantitative research checklists

Evaluation Tool for Quantitative Research Studies

Building on work within a project exploring the feasibility of undertaking systematic reviews of research literature on effectiveness and outcomes in social care, a set of evaluation tools have been developed to assist in the critical appraisal of research studies. The evaluation tool for quantitative studies contains six sub-sections: study evaluative overview; study, setting and sample; ethics; group comparability and outcome measurement; policy and practice implications; and other comments. It provides a template of key questions to assist in the critical appraisal of quantitative research studies.

Review Area	Key Questions
**(1) STUDY OVERVIEW**	
Bibliographic Details	0. Author, title, source (publisher and place of publication), year
Purpose	1. What are the aims of the study?<br>2. If the paper is part of a wider study, what are its aims?
Key Findings	3. What are the key findings of the study?
Evaluative Summary	4. What are the strengths and weaknesses of the study and theory, policy and practice implications?
**(2) STUDY, SETTING, SAMPLE AND ETHICS**	
The Study	5. What type of study is this?<br>6. What was the intervention?<br>7. What was the comparison intervention?<br>8. Is there sufficient detail given of the nature of the intervention and the comparison intervention?<br>9. What is the relationship of the study to the area of the topic review?
Setting	10. Within what geographical and care setting was the study carried out?
Sample	11. What was the source population?<br>12. What were the inclusion criteria?<br>13. What were the exclusion criteria?<br>14. How was the sample selected?<br>15. If more than one group of subjects, how many groups were there, and how many people were in each group?<br>16. How were subjects allocated to the groups?<br>17. What was the size of the study sample, and of any separate groups?<br>18. Is the achieved sample size sufficient for the study aims and to warrant the conclusions drawn?<br>19. Is information provided on loss to follow up?<br>20. Is the sample appropriate to the aims of the study?<br>21. What are the key sample characteristics, in relation to the topic area being reviewed?
**(3) ETHICS**	
Ethics	22. Was Ethical Committee approval obtained?<br>23. Was informed consent obtained from participants of the study?<br>24. Have ethical issues been adequately addressed?

**(4) GROUP COMPARABILITY AND OUTCOME MEASUREMENT**	
Comparable Groups	25. If there was more than one group was analysed, were the groups comparable before the intervention? In what respects were they comparable and in what were they not?<br>26. How were important confounding variables controlled (e.g. matching, randomisation, in the analysis stage)?<br>27. Was this control adequate to justify the author's conclusions?<br>28. Were there other important confounding variables controlled for in the study design or analyses and what were they?<br>29. Did the authors take these into account in their interpretation of the findings?
Outcome Measurement	30. What were the outcome criteria?<br>31. What outcome measures were used?<br>32. Are the measures appropriate, given the outcome criteria?<br>33. What other (e.g. process, cost) measures are used?<br>34. Are the measures well validated?<br>35. Are the measures of known responsive to change?<br>36. Whose perspective do the outcome measures address (professional, service, user, carer)?<br>37. Is there a sufficient breath of perspective?<br>38. Are the outcome criteria useful/appropriate within routine practice?<br>39. Are the outcome measures useful/appropriate within routine practice?
Time Scale of Measurement	40. What was the length of follow-up, and at what time points was outcome measurement made?<br>41. Is this period of follow-up sufficient to see the desired effects?
**(5) POLICY AND PRACTICE IMPLICATIONS**	
Implications	42. To what setting are the study findings generalisable? (For example, is the setting typical or representative of care settings and in what respects?)<br>43. To what population are the study's findings generalisable?<br>44. Is the conclusion justified given the conduct of the study (For example, sampling procedure; measures of outcome used and results achieved?)<br>45. What are the implications for policy?<br>46. What are the implications for service practice?
**(6) OTHER COMMENTS**	
Other Comments	47. What were the total number of references used in the study?<br>48. Are there any other noteworthy features of the study?<br>49. List other study references
Reviewer	50. Name of reviewer<br>51. Review date

Source: Long AF, Godfrey M, Randall T, Brettle AJ and Grant MJ (2002) *Developing Evidence Based Social Care Policy and Practice. Part 3: Feasibility of Undertaking Systematic Reviews in Social Care*. Leeds: Nuffield Institute for Health.

Note: This tool was developed while the lead author was at the Health Care Practice R&D Unit (HCPRDU) at the University of Salford. It has since been slightly modified.

Appendice 4 Health Care Practice R&D Unit (HCPRDU) mixed methods research checklists

Evaluation Tool for 'Mixed Methods' Study Designs

The 'mixed method' evaluation tool was developed from the evaluation tools for 'quantitative' and 'qualitative' studies,[i] themselves created within the context of a project exploring the feasibility of undertaking systematic reviews of research literature on effectiveness and outcomes in social care. The 'mixed method' tool draws on appropriate questions from the quantitative and qualitative evaluation tools. It provides a template of key questions to assist in the critical appraisal of studies using more than one method.[ii]

Review Area	Key Questions
**(1) STUDY EVALUATIVE OVERVIEW**	
Bibliographic Details	• Author, title, source (publisher and place of publication), year
Purpose	• What are the aims of this paper?<br>• If the paper is part of a wider study, what are its aims?
Key Findings	• What are the key findings?
Evaluative Summary	• What are the strengths and weaknesses of the study and theory, policy and practice implications?
**(2) STUDY AND CONTEXT (SETTING, SAMPLE AND OUTCOME MEASUREMENT)**	
The Study	• What type of study is this?<br>• What was the intervention?<br>• What was the comparison intervention?<br>• Is there sufficient detail given of the nature of the intervention and the comparison intervention?<br>• What is the relationship of the study to the area of the topic review?
Context: (1) Setting	• Within what geographical and care setting is the study carried out?<br>• What is the rationale for choosing this setting?<br>• Is the setting appropriate and/or sufficiently specific for examination of the research question?<br>• Is sufficient detail given about the setting?<br>• Over what time period is the study conducted?
Context II: Sample	• What was the source population?<br>• What were the inclusion criteria?<br>• What were the exclusion criteria?<br>• How was the sample (events, persons, times and settings) selected? (For example, theoretically informed, purposive, convenience, chosen to explore contrasts)<br>• Is the sample (informants, settings and events) appropriate to the aims of the study?<br>• If there was more than one group of subjects, how many groups were there, and how many people were in each group?<br>• Is the achieved sample size sufficient for the study aims and to warrant the conclusions drawn?<br>• What are the key characteristics of the sample (events, persons, times and settings)?
Context III: Outcome Measurement	• What outcome criteria were used in the study?<br>• Whose perspectives are addressed (professional, service, user, carer)?<br>• Is there sufficient breadth (e.g. contrast of two or more perspective) and depth (e.g. insight into a single perspective)?

Review Area	Key Questions
**(3) ETHICS**	
Ethics	• Was Ethical Committee approval obtained?<br>• Was informed consent obtained from participants of the study?<br>• How have ethical issues been adequately addressed?
**(4) GROUP COMPARABILITY**	
Comparable Groups	• If there was more than one group was analysed, were the groups comparable before the intervention? In what respects were they comparable and in what were they not?<br>• How were important confounding variables controlled (e.g. matching, randomisation, or in the analysis stage)?<br>• Was this control adequate to justify the author's conclusions?<br>• Were there other important confounding variables controlled for in the study design or analyses and what were they?<br>• Did the authors take these into account in their interpretation of the findings?
**(5) QUALITATIVE DATA COLLECTION AND ANALYSIS**	
Data Collection Methods	• What data collection methods were used in the study? (Provide insight into: data collected, appropriateness and availability for independent analysis)<br>• Is the process of fieldwork adequately described? (For example, account of how the data were elicited; type and range of questions; interview guide; length and timing of observation work; note taking)
Data Analysis	• How were the data analysed?<br>• How adequate is the description of the data analysis? (For example, to allow reproduction; steps taken to guard against selectivity)<br>• Is adequate evidence provided to support the analysis? (For example, includes original / raw data extracts; evidence of iterative analysis; representative evidence presented; efforts to establish validity - searching for negative evidence, use of multiple sources, data triangulation); reliability / consistency (over researchers, time and settings; checking back with informants over interpretation)<br>• Are the findings interpreted within the context of other studies and theory?
Researcher's Potential Bias	• What was the researcher's role? (For example, interviewer, participant observer)<br>• Are the researcher's own position, assumptions and possible biases outlined? (Indicate how these could affect the study, in particular, the analysis and interpretation of the data)

Review Area	Key Questions
**(6) POLICY AND PRACTICE IMPLICATIONS**	
Implications	• To what setting are the study findings generalisable? (For example, is the setting typical or representative of care settings and in what respects? If the setting is atypical, will this present a stronger or weaker test of the hypothesis?)<br>• To what population are the study's findings generalisable?<br>• Is the conclusion justified given the conduct of the study (For example, sampling procedure; measures of outcome used and results achieved?)<br>• What are the implications for policy?<br>• What are the implications for service practice?
**(7) OTHER COMMENTS**	
Other comments	• What was the total number of references used in the study?<br>• Are there any other noteworthy features of the study?<br>• List other study references
Reviewer	• Name of reviewer<br>• Review date

---

[i] Long AF, Godfrey M, Randall T, Brettle AJ and Grant MJ (2002) *Developing Evidence Based Social Care Policy and Practice. Part 3: Feasibility of Undertaking Systematic Reviews in Social Care*. Leeds: Nuffield Institute for Health.

[ii] This tool was developed while the lead author was at the Health Care Practice R&D Unit (HCPRDU) at the University of Salford. It has since been slightly modified.

Appendice 5 Example of using CASP RCT checklist to appraise a selected RCT

Bibliographic Details: Emery, et al., ProCare Trial: a phase II randomized controlled trial of shared care for follow-up of men with prostate cancer, BIU International, Australia, 2017 [28]	
Screening questions	Score and reason
Did the trial address a clearly focused issue?<br>Consider: An issue can be 'focused' In terms of<br>• The population studied<br>• The intervention given<br>• The comparator given<br>• The outcomes considered	Score: 2<br>This study addressed a focused question. First, there were clear inclusion and exclusion criteria for the study population. Second, the study procedures were also very clear and detailed in both intervention group and control group. Third, the trial reported all outcomes clearly and the questionnaires were suitable for the research purpose.
2. Was the assignment of patients to treatments randomised?<br>Consider:<br>• How was this carried out, some methods may produce broken allocation concealment<br>• Was the allocation concealed from researchers?	Score: 2<br>The randomization was conducted by an independent professional centre after both patients and their GPs had signed informed consent, so the allocation was concealed from researchers. Although as mentioned in the paper, there might be potential selection bias because the relationship between patients and practitioners could affect the patients' decision about participation. However, the author also provide a reasonable explanation so that the rigor of the study is not affected.
3. Were patients, health workers and study personnel blinded?<br>Consider:<br>• Health workers could be; clinicians, nurses etc<br>• Study personnel – especially outcome assessors	Score: 0<br>As for this research, it is impossible to blind patients and practitioners because they are aware of the whole process. But the study did not mentioned about keeping outcome assessors blinded, which is a shortage of this research.
4. Were the groups similar at the start of the trial?<br>Consider: Look at<br>• Other factors that might affect the outcome such as age, sex, social class, these may be called baseline characteristics	Score: 2<br>The important baseline factors such as age, stage of cancer, and treatment type had been considered and well balanced. The author also stratified the allocation in the randomization since patients came from different treatment centres.
5. Aside from the experimental intervention, were the groups treated equally?	Score: 1<br>This study had considered about balancing two arms. All patients received five follow-up visits, the difference between two group was two specialist follow-up replaced by the GPs. The only difference was the GPs visited the patients at the beginning to re-engage with them, which might affect the results.
6. Were all of the patients who entered the trial properly accounted for at its conclusion?<br>Consider:<br>• Was the trial stopped early?<br>• Were patients analysed in the groups to which they were	Score: 2<br>No evidence showed the trial stopped early. All patients with results had been analysed properly.

randomised?	
7. How large was the treatment effect?<br>Consider:<br>• What outcomes were measured?<br>• Is the primary outcome clearly specified?<br>• What results were found for each outcome?<br>• Is there evidence of selective reporting of outcomes?	Score: 2<br>All outcome measurements were patient-reported questionnaires. The reliability and validity had been discussed in the research protocol. The primary outcomes included four questionnaire, a single specific question about patients' preference of care model, and a financial assessment. All results had been reported clearly, and no significant difference between two groups expect the single specific question (P<0.001). Besides, no evidence indicated selective reporting.
8. How precise was the estimate of the treatment effect?<br>Consider:<br>• What are the confidence limits?<br>• Were they statistically significant	Score: 2<br>The 95% confidence interval were provided in the study, and the sample size could provide 80% power to detect differences of 0.6 SD at two side. The significant differences were defined as P<0.05.
9. Can the results be applied in your context? (or to the local population?)<br>Consider:<br>• Do you have reason to believe that your population of interest is different to that in the trial<br>• If so, in what way?	Score: 1<br>Although the design of this research was rigorous and clear, the system is different from China. However, some developed area in China begin to pay attention to primary care, and this shared care model could be a good model in the future.
10. Were all clinically important outcomes considered?<br>Consider:<br>• Is there other information you would like to have seen?<br>• Was the need for this trial clearly described?	Score: 1<br>Most important information has been reported, and the needs also clearly described. The limit was the number of patients changed in the outcome report table, but the author did not explain the reason. Besides, the article did not mentioned how to deal with incomplete questionnaires.
11. Are the benefits worth the harms and costs?<br>Consider:<br>• Even if this is not addressed by the trial,<br>• what do you think?	Score: 2<br>Since the results in this research showed that shared care could provide similar outcomes to usual care with lower costs, the benefit worth all the efforts.
Total	Score: 17

"0" represents many limitations, "1" represents some limitation, "2" represents excellent.

Appendice 6 Example of using HCPRDU quantitative research checklists to appraise a selected quantitative study

Bibliographic Details: Lund, Shared Care in prostate cancer: a three-year follow-up, SCANDINAVIAN JOURNAL OF UROLOGY, Denmark, 2016 [35]	
Review Area	Score and Reason
(1) STUDY OVERVIEW	Score: 1<br>This is a multicentre research which lasted for three years. The bibliographic details were provided, and the key finding was clearly defined. However, the weaknesses are the definition of “shared care” was not detailed and the objective of this study was not clear.
(2) STUDY, SETTING, SAMPLE AND ETHICS	Score: 1<br>The limitation is the research type was not mentioned in the content, and there was no comparison intervention. Besides, whether the sample size was sufficient has not been defined, which could not warrant the conclusions drawn.<br>The study was based on the shared care model, and the patient discharge summery proceeded as the standard of follow-up recommendation, which was provided to the GPs. The inclusion and exclusion criteria were described. The patients were enrolled from three hospitals and they were stratified according to hospital, age, and treatment, the dropout was also distributed to three hospitals, which could warrant the conclusion. Besides, all the outcomes were illustrated clearly as well as how to deal with the missing data.
(3) ETHICS	Score: 1<br>The paper did not mention about the ethical approval, but all patients had given their consent.
(4) DATA COLLECTION, ANALYSIS AND POTENTIAL RESEARCHER BIAS	Score: 1<br>The author had considered about the confounding variables when analysing the data. The outcome measures were appropriate in the study. All data was collected and provided the sufficient details about the meaning. Besides, the length of follow-up was three years, which was sufficient to detect the effects. Although the non-responders could lead to some bias, the dropout was equally distributed in terms of hospital and age, and there was no difference between non-responders and participants.<br>The limit is that there was no comparison and the validity and reliability of questionnaires were not clarified, which might affect the credibility of the conclusion.
(5) POLICY AND PRACTICE IMPLICATIONS	Score: 2<br>The study findings underlined shared care could increase the patient and GP compliance in cancer follow up, which could be widely utilised in practice.
(6) OTHER COMMENTS	Score: 2<br>The references were listed in the paper. In generally, it provided a feasible way of follow up for cancer patients based on a multicentre and long term research.
Total	Score: 8

GP=general practices

“0” represents many limitations, “1” represents some limitation, “2” represents excellent.

Appendice 7 Example of using HCPRDU mixed methods research checklists to appraise a selected mixed methods' study

Question	Score
	Bibliographic Details: Hanan, et al., Delivering care to oncology patients in the community: an innovative integrated approach, INNOVATIONS IN CARE, Ireland, 2014 [24]
(1) STUDY OVERVIEW	Score: 1<br>The aim and key findings were described in the text. The strengths of the study is that the intervention was innovative and clearly defined, which provide a new way to carry out shared care between hospital nurses and community nurses. The weakness was that the quantitative outcome measurements and results were not clearly specified, and no data was reported.
(2) STUDY AND CONTEXT (SETTING, SAMPLE AND OUTCOME MEASUREMENT)	Score: 1<br>This study is mixed type study and the intervention was quite clear and the design is very creative. There is adequate detail in intervention group and comparison group. The rationale for the study programme was explained and the shared care was home-cased care majorly conducted by community nurses. As for sample, the author did not mentioned about how many participants were involved in the quantitative procedure. Besides, the outcome measurement was not mentioned in the text.
(3) ETHICS	Score: 1<br>The study obtained ethical approval and oral informed consent was provided to the patients who joined the qualitative interview. But whether the participants signed informed consent was not mentioned.
(4) GROUP COMPARABILITY	Score: 0<br>The author mentioned the data form three distinct time points would be collected and compared to hospital activity data, but no relevant data could be found in the paper.
(5) QUALITATIVE DATA COLLECTION AND ANALYSIS	Score: 1<br>The data collection methods were provided, but the process of fieldwork such as the interview guide and length was not adequately described. The description of data analysis process was mentioned but not sufficient. The findings were quite clear. The interview and the coding process were conducted by the researcher team, so there might be some potential bias.
(6) POLICY AND PRACTICE IMPLICATIONS	Score: 1<br>The cancer survivors are increasing in the whole world. The nurses play an important role in the management of cancer patients. This research provide a good way to combine oncology nurses and community nurses in the integrated care. However, the programme might cost a lot of money, which is the major obstacle of implementation.
(7) OTHER COMMENTS	Score: 1<br>Although the design of the study was quite good in this mixed study, but more details about the data collection and data analysis in both quantitative and qualitative part

	should be covered.
Total score (maximum 14)	6

"0" represents many limitations, "1" represents some limitation, "2" represents excellent
